# Application of probiotics in adjuvant treatment of infant allergic rhinitis: A randomized controlled study

**DOI:** 10.1097/MD.0000000000020095

**Published:** 2020-05-01

**Authors:** Xueqiu Cao, Ping Zhong, Gang Li, Jiao Zhu, Yun Zheng

**Affiliations:** a Hearing Center/Hearing and Speech Science Laboratory, Department of Otolaryngology Head and Neck Surgery, West China Hospital of Sichuan University, Chengdu; b Department of Otorhinolaryngology-Head and Neck Surgery, Minda Hospital of Hubei Minzu University, Enshi, China.

**Keywords:** allergic rhinitis, infants, prebiotics, randomized controlled trial, young children

## Abstract

**Background:**

Several studies have suggested that intestinal flora may play an important role in allergic diseases. The purpose of this study was to investigate the effect of probiotics of Bifidobacterium triplex on the symptoms of allergic rhinitis (AR). The effects of this probiotic on the immune system have been reported in some studies, but most previous studies have been in animals.

**Methods/Design:**

60 infants and young children with AR were randomly divided into 2 groups: probiotics/placebo group. The main outcome was the use of a comprehensive symptom drug score to assess allergy symptoms. In addition, health-related quality of life was investigated (rhinitis quality of life questionnaire). Secondary outcomes included a visual analog scale of allergy burden and a second quality of life questionnaire. This report describes the study design of a randomized controlled trial.

**Discussion:**

The study design described a double-center, randomized, location at the Minda Hospital of Hubei Minzu University and West China Hospital of Sichuan University, which will be focused on the study about probiotics treatment and its effect on AR symptoms.

**Trial registration:**

It has been registered at http://www.chictr.org.cn/listbycreater.aspx (Identifier: ChiCTR2000031175), Registered March 22, 2020.

## 1 Introduction

Allergic rhinitis (AR) is a non-infectious inflammatory disease of nasal mucosa mediated by specific IgE after exposure to allergens in susceptible infants and young children.^[1]^ AR is mainly characterized by clinical manifestations such as clear watery nose and nasal congestion, which can be accompanied by eye symptoms and ear symptoms. AR is life-threatening, however it can significantly affect the quality of life of infants and young children, leading to poor sleep quality and lack of concentration, which seriously affects the growth and development of infants and young children. In addition, AR is also closely related to asthma, nasal polyps, sinusitis, otitis media and other diseases. The prevalence of AR has been increasing year by year globally, ranging from 2.2% to 45.1%.^[2]^ With the rapid development of society and changes in living environment, the incidence of AR among infants and young children in China is also increasing, which seriously affects the physical and mental health of infants and young children.^[3]^

In recent years, Sakaguchi et al found that the TH_1_ / TH_2_ immune response imbalance of CD4 + T lymphocytes plays an important role in the pathogenesis of AR. This theory is to discuss the pathogenesis of AR from the perspective of immunology.^[4]^ The strong TH_2_ cell reaction plays an important role in the occurrence and development of AR.

Probiotics and breast milk are identified as two factors that can affect the course of allergic disease. The effect of probiotics on immune function has been a hot topic in allergic diseases in recent years. It has been reported that probiotics can improve the intestinal mucosal immune function of allergic rats and down-regulate the function of TH_2_ cytokines to mediate the TH_1_/ TH_2_ balance, thus preventing the occurrence of food allergy. Animal experiments have found that lactic acid bacteria can promote TH_1_ immune response and inhibit TH_2_ immune response, so as to put the body's immune system in a dynamic balance and inhibit the occurrence of allergic reactions.^[5]^

Prebiotics are defined by Gibson and Roberfroid, first proposed in 1995: Prebiotics are undigested or indigestible food ingredients that contribute to host health by selectively stimulating bacterial proliferation and/or activity in the colon. Some studies have found that breastfeeding within 6 months after birth is a protective factor for AR in infants and young children, and Odijk et al^[6]^ found that breastfeeding for more than 3 months can effectively reduce the probability of infants suffering from ectopic dermatitis and laryngeal wheezing, especially for those infants and young children with a family history of allergies. One of the reasons that breast-fed infants are less susceptible to allergic diseases than artificially fed infants is the presence of beneficial oligosaccharides in breast milk. Bifidobacteria dominated the intestinal flora of breast-fed infants at 6 to 7 days, while the number of bifidobacteria in the intestinal flora of artificially fed infants was small and the bacterial species was diverse. In present study, we aim to conduct a double-blind randomized controlled trial study to explore the effect of probiotics on AR in infants and young children.

## 2 Methods/design

### 2.1 Research object

2.1.1 We aim to explore the role of probiotics in preventing allergic diseases in infants and young children.

2.1.2 We aim to explore the effect of probiotics on AR in infants and young children.

### 2.2 Study method

A total of 60 patients with AR in Otorhinolaryngology-Head and Neck Surgery, Minda Hospital of Hubei Minzu University and West China Hospital of Sichuan University from May 2020 to April 2022 will be selected and randomly divided into probiotic/placebo group according to the numerical table. The study divided the patients into two groups. Patients will be divided into a double-blind probiotic/placebo study, we will assess allergy Symptoms using health-related quality of life by means of diaries, quality of life problems (RQLQ), and Combined Symptoms and Medication Score (CSMS). CSMS is a simple and standardized way to balance symptoms with the need for antidepressants. RQLQ is a disease-specific indicator used to assess health-related quality of life, including the patient's physical, social and emotional health.

### 2.3 Participants

Inclusion criteria include the following:

(1) We will recruit and include 60 participants aged 0 to 6 with a history of AR.
(2) The plan has an equal proportion of male and female, however this is not enforced.
(3) AR must be diagnosed by a doctor (allergist or general practitioner).
(4) Participants were required to present the results of tests sensitive to air allergen IgE (either skin prick test or serum allergen-specific IgE) no more than 24 months prior to the trial.

Exclusion criteria include the following:

Patients with a history of diabetes, gastrointestinal disease, use of antibiotic drugs in the past 6 weeks, and any known psychiatric or neurological disorders.

Anatomical changes such as septal rupture or perforation were also ruled out.

### 2.4 Interventions

The study design will be described as a two-center, randomized, location study of probiotics in affiliated hospital of Hubei university for nationalities and its effect on AR symptoms. Probiotic treatment will be compared to the placebo application model, and we will provide probiotics to all patients for another 4 weeks after the trial phase.

Probiotic treatment will be performed by Bifidobacterium triplex. Probiotics are provided in drops. The placebo treatment consisted of dropping carrier solutions containing probiotic treatments (lactose monohydrate, glucose monohydrate), but was no different in color, smell, or taste from probiotics.

### 2.5 Data collection: quality management and storage

Reservations will be made at the end of the first meeting for the following dates to facilitate participants’ reservations. Data from each measurement will be collected in writing at a face-to-face meeting and then recorded electronically at the Minda Hospital of Hubei Minzu University. Once recorded, the data is locked to prevent changes. Data lost because it did not appear will be encoded as incomplete. Then, SPSS vision. 25 will be used to analyze the result data. All data collected on the paper will be tagged with a research identification number to prevent identification of participants and stored in a locked cabinet. Only the author of the research report can access these identified data sets.

### 2.6 Measurement of outcomes

Tools to measure primary indicators include the following:

(1) The health-related quality of life by means of diaries.
(2) In this study, RQLQ will be used to evaluate the comfort level of the 2 groups of patients.
(3) CSMS will be used to evaluate allergy symptoms.

### 2.7 Blinding and random

After completing the first evaluation (the first interview), the research assistant opened an opaque envelope to determine that the participants were randomly assigned to two groups. Randomization is based on a computer-generated sequence of random Numbers built by an independent investigator. These researchers will be independent of the study members responsible for recruiting participants. All outcome measurements will be performed by blind experimenters.

### 2.8 Statistical analysis

Statistical analyses will be implemented by SPSS 17.0 and Microsoft Excel 2007 software. Data will be represented as mean ± standard deviation (SD). A *t*-test will be performed to compare the changes in measures within groups. Statistical significance will be considered at *P* < .05.

## 3 Discussion

The effect of probiotics on immune function has been a hot topic in allergic diseases in recent years.^[7,8]^ In recent years, it has been widely reported that probiotics can improve the intestinal mucosal immune function of allergic rats and down-regulate the function of TH_2_ cytokines to mediate the TH_1_/ TH_2_ balance, thus preventing the occurrence of food allergy.^[9]^ Animal experiments have revealed that lactic acid bacteria can promote TH_1_ immune response, inhibit TH_2_ immune response, make the body's immune system in a dynamic balance and inhibit the occurrence of allergic reactions. In addition, animal studies have demonstrated that lactic acid bacteria can induce the production of IL-12 and IFN-γ, reduce the production of IL -4, IL-5 and IL-3, promote the immune response of TH_1_ and inhibit the occurrence of allergic reactions. Therefore, prebiotics might have the preventing potential on AR.

Prebiotics are a group of indigestible food ingredients that promote the colonization and growth of probiotics in the intestinal environment.^[10,11]^ Probiotics can also secrete TGF-β and interleukin-10 through TH_3_ cells and Tr1 cells, inhibit the production of Ig E, thus inhibiting the TH_2_ immune response, so as to put the immune system in a dynamic equilibrium state.^[12]^ In recent years, studies have found that infants and young children born after six months of breast-feeding is the protective factors of AR infants and young children breastfed babies than formula-fed infants are less likely to suffer from allergic diseases,^[13]^ one of the reasons is beneficial in breast milk is born yuan oligosaccharides or low poly galactose, after the birth of the baby to play an important role in the development of the immune system.^[14,15]^

The study design described a double-center, randomized, location at the Minda Hospital of Hubei Minzu University and West China Hospital of Sichuan University, which will be focused on the study about probiotics treatment and its effect on AR symptoms.

## Author contributions

Xueqiu Cao conceived the idea for this study; Ping Zhong and Jiao Zhu provided statistical plan; Xueqiu Cao and Gang Li drafted the protocol. Yun Zheng reviewed the protocol and provided critical feedback. All authors approved the article in its final form.
